# Presenteeism and absenteeism in the manufacturing sector: A multilevel approach identifying underlying factors and relations to health

**DOI:** 10.1007/s12144-022-03013-1

**Published:** 2022-04-02

**Authors:** Joshua Nowak, Andre Emmermacher, Johannes Wendsche, Antonia-Sophie Döbler, Jürgen Wegge

**Affiliations:** 1 Present Address: Faculty of Psychology, TU Dresden, Dresden, Germany; 2 Present Address: Department of Product, Grover Group GmbH, Berlin, Germany; 3 Present Address: Siemens Energy Global GmbH & Co.KG, Berlin/München, Germany; 4 Federal Institute for Occupational Safety and Health, Fabricestr 8, 01099 Dresden, Germany; 5 Present Address: Department of People Development, Bechtle AG, Neckarsulm, Germany

**Keywords:** Absenteeism, Context strength, Core self-evaluations, Health, Multilevel, Presenteeism

## Abstract

Presenteeism is problematic since it relates to lower health and productivity. Prior research examined many work and attitudinal variables relating to presenteeism at the individual level. Here, we conceptualize presenteeism as multilevel phenomenon also shaped by the overall attendance behavior (absenteeism and presenteeism) at the work unit. We surveyed employees at a manufacturing plant on presenteeism, health-related lost productive time (HLPT) and absenteeism (*N* = 911, 22 units) and collected preceding (past 12–7 and 6 months) objective absence data aggregating it at unit level. Considering the individual-level antecedents only higher physical demands predicted higher absence duration. Presenteeism related positively to physical demands, a burdensome social environment, and organizational identification and negatively to ease of replacement, and core self-evaluations. These relationships were similar for HLPT as outcome. Regarding unit-level factors, preceding unit-level absence frequency (but not duration) negatively related to presenteeism. The negative relationship between core self-evaluations and individual presenteeism decreased under a stronger presenteeism context supporting the hypothesized cross-level effect of unit-level presenteeism context strength. Moreover, individual and unit-level presenteeism correlated, as expected, more strongly with health complaints than absenteeism. Our study demonstrates the value of a contextual, multilevel approach for understanding antecedents and consequences of attendance behavior.

## Introduction

Sick employees make a critical choice between absence and attendance at work, leading to important individual and organizational consequences. Whereas the first option, sickness *absenteeism*, has a long tradition in research (Muchinsky, 1977), the latter option, *presenteeism—*commonly defined as attending work while sick*—*only gained broader attention within the last two decades (Johns, 2010; Ruhle et al., 2020). The growing interest in this behavior roots in several observations. For instance, it was found that presenteeism relates stronger to health than absenteeism (Aronsson et al., 2011). This can be expected when periods of health impairment are more often spent as presenteeism than absenteeism, i.e. for preventing high absenteeism rates at work (Gerich, 2015). Hence, ignoring this phenomenon and focusing organizational efforts of health and HR management solely on lowering absence rates can come at the cost of high presenteeism (Caverley et al., 2007). High presenteeism, in turn, can also affect the future health of employees by impairing their own recuperation (Demerouti et al., 2009) and enhancing contagion of health impairments among coworkers (Lovell, 2004). Moreover, managing presenteeism is also important to prevent the high costs of associated productivity loss occurring when employees work though they are sick (Hemp, 2004). It was found that these costs can even exceed those of absenteeism (Collins et al., 2005). Unfortunately, only few prior studies simultaneously measured presenteeism and associated productivity losses (Johns, 2011), also because presenteeism was sometimes defined as the productivity loss that occurs when working while sick (Johns, 2010).

Accordingly, future research on presenteeism should differentiate between presenteeism behavior as such and productivity-focused indices like health-related lost productive time (HLPT) that assess the performance impairment *resulting* from working while sick (Brooks et al., 2010; Pohling et al., 2016). Another, related problem of prior research is that absenteeism was frequently neglected in studies examining the emergence and consequences of presenteeism (Johns, 2010), concealing the potentially diverging impacts of work-related or personal factors on both types of behavior. For instance, although absenteeism and presenteeism correlate often positively (Miraglia & Johns, 2016), their antecedents and consequences are likely different (Böckerman & Laukkanen, 2010; Schmidt et al., 2019). Thus, the closely related concepts of presenteeism, absenteeism, and potential productivity losses due to presenteeism need to be studied in conjunction. Moreover, we argue in this paper that prior research has mostly focused on individual-level factors associated with presenteeism. Whereas the multilevel context in analyzing absenteeism is acknowledged for many years (e.g., see Diestel et al., 2014), this perspective is not yet common in presenteeism research (Ruhle et al., 2020). Therefore, the role of contextual variables at the work unit level is not well understood. For instance, there is little knowledge on the relation between individual and unit-level presenteeism. To further advance these research developments, we approach this study with a multilevel perspective. We examine the association between preceding objective unit-level absenteeism and individual presenteeism. Moreover, we analyze presenteeism as a contextual factor at the work unit level and investigate how presenteeism context strength affects the relationship between a trait variable and presenteeism on the individual level. To complement these unit-level analyses, we investigate a set of individual-level variables that includes both well-researched factors underlying attendance behavior as well as further variables based on theoretical considerations. Additionally, we examine employees’ mental *and* physical health since presenteeism relates to both (Gerich, 2015; Johns, 2010).

## Theoretical Background

### Factors Underlying Attendance Behavior and HLPT at the Individual Level

**Fig. 1:**
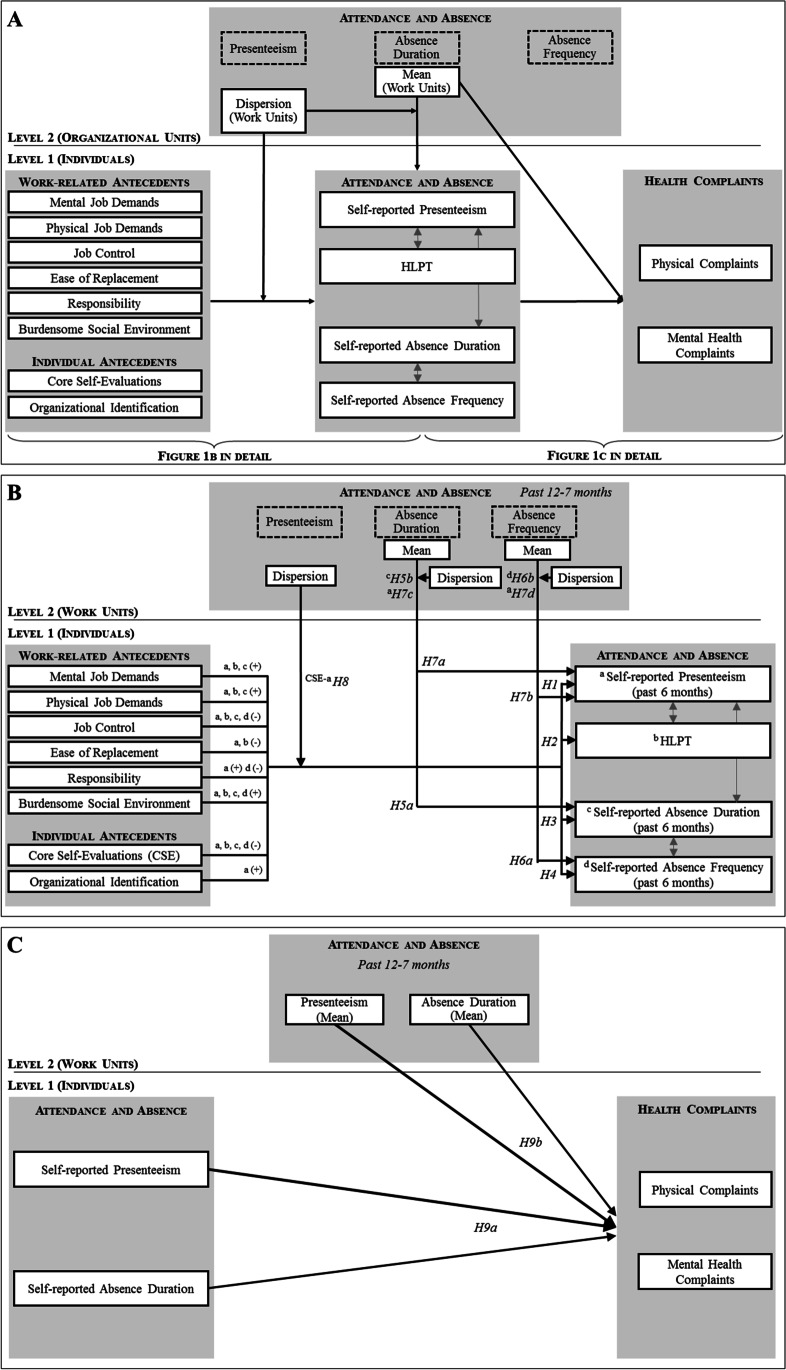
A Multilevel Model on Attendance and Absence Behavior and Health Complaints. *Note*. A: Condensed research model, B: Hypothesized research model with multilevel relationships between anteceding factors and attendance/absence behavior, C: Hypothesized research model with multilevel relationships between attendance/absence behavior and health complaints (stronger bold arrows representing stronger relationships)

Researchers have identified many work-related and personal factors associated with presenteeism (Miraglia & Johns, 2016) but only few studies conjointly examined their relation to absenteeism or productivity loss (Johns, 2011). We decided to conduct such a conjoint analysis. To this end, we chose a set of variables (Fig. 1A and B) that includes both well-researched, meta-analytically studied factors as well as novel variables based on theoretical considerations described below. In doing so, we aim to both corroborate and expand on the understanding of individual-level factors underlying attendance behavior and productivity.

*Job demands* are defined as physical, psychological, social, or organizational job characteristics that need effort and result in psychophysical costs (Bakker et al., 2003). Their relation with presenteeism is small and positive (Miraglia & Johns, 2016). Correlations between job demands and absenteeism are also small and positive (for absence duration) but negligible for absence frequency (Schaufeli et al., 2009).

*Job control* is a job resource that describes employee’s influence on their own work (Bakker et al., 2003). Job resources strengthen work engagement and decrease burnout, thus reducing absence duration and frequency (Schaufeli et al., 2009). Concerning presenteeism, job control has sometimes been conceived as “adjustment latitude”, reflecting the extent to which employees can make adaptations to their work when their health is impaired (Johansson & Lundberg, 2004). Aronsson and Gustafsson (2005) noted that the relationship between job control and presenteeism can vary. It might be *positive* since adapting tasks according to health-related performance impairments allows employees to attend work despite sickness or *negative* given the positive relation of job control and health (Bond & Bunce, 2003). Thus, some studies found nonexistent (Miraglia & Johns, 2016) or even curvilinear (Gerich, 2019) relationships. We assume to find a negative relationship in our study for two reasons. First, the ability of health-related work adjustment does not imply that employees exploit this option, whereas an improvement of health (through higher job control) should inevitably lead to less presenteeism. Second, in our research setting (production work) low autonomy prevails regarding timing, location or method of working. Finally, HLPT could also be affected by job control since productivity increases with higher job control (Bond & Bunce, 2003), yet it is unclear whether this is attributable to health.

*Ease of replacement* was often studied as a factor negatively related to presenteeism, commonly operationalized as the amount of work left undone after a period of absenteeism (Aronsson & Gustafsson, 2005). At this, scholars understood higher amounts of work left undone as a sign of lower replaceability. Since this approach might not be suitable to work that does not allow a delay of tasks, this study employs a broader operationalization, which directly prompts employees to estimate their ease of replacement (Johns, 2011). A meta-analysis found ease of replacement to be negatively related with presenteeism (Miraglia & Johns, 2016) whereas the relation to productivity loss is smaller and a relation to absenteeism does not seem to exist (Johns, 2011).

Job *responsibility* was studied as an antecedent of attendance behavior as well. Past research revealed that employees with a higher hierarchical level show less absenteeism (Bierla et al., 2013) whereas more cooperation (Hansen & Andersen, 2008) and feelings of team-related social obligation (Grinyer & Singleton, 2000) increase presenteeism. We assume that such findings relate to perceptions of job responsibility as an underlying factor.

Similarly, the quality of the *social environment*, such as collegial relationships and support, was studied in association with presenteeism (Miraglia & Johns, 2016). This variable likely affects the feelings of social obligation that sometimes underlie presenteeism (Grinyer & Singleton, 2000) and make employees more inclined to avoid absenteeism in order to prevent the social environment from further deterioration. At the same time, a burdensome social environment could also be understood as a hindrance demand that promotes withdrawal behavior such as absenteeism (Podsakoff et al., 2007).

In addition to the largely well-understood variables that we included for replication purposes, we examine two further concepts based on the following theoretical considerations. First, *core self-evaluations* (CSE) are a personality construct encompassing the traits self-esteem, self-efficacy, locus of control, and neuroticism (Judge et al., 2003). Johns (2011) found that (health-related) locus of control as one component of CSE negatively relates to presenteeism. We further assume that CSE negatively relates to absence since psychological capital, including self-efficacy as subcomponent, negatively relates to absence (Avey et al., 2006). Moreover, job performance, a positive correlate of CSE (Judge & Bono, 2001), negatively relates to absence (Viswesvaran, 2002). In addition, CSE positively relates to physical and mental health (Tsaousis et al., 2007). Taken together, we hypothesize that CSE is an important personality disposition that negatively relates to presenteeism and absenteeism.

The second variable is *organizational identification* (OID), which reflects “the perception of oneness with […] an organization, where the individual *defines* him or herself in terms of the organization(s) in which he or she is a member” (Mael & Ashforth, 1992, p. 104). To our knowledge, previous research on presenteeism did not consider OID*.* While OID is unrelated to absenteeism (Riketta, 2005), it positively correlates with health (Steffens et al., 2016). In our study, we argue that a positive correlation between OID and presenteeism is to be expected for two reasons. First, OID is associated with organizational citizenship behavior (Feather & Rauter, 2004) and presenteeism can be clearly viewed as such a behavior (Johns, 2010). Second, since highly identified employees define themselves through the organization’s successes (Mael & Ashforth, 1992), they are likely more prone to tolerate minor health impairments at work in order to continue contributing to the organization’s success.

Finally, facets of absenteeism and presenteeism often correlate positively with each other, for instance, absence duration with absence frequency (Bakker et al., 2003) and presenteeism (Miraglia & Johns, 2016), and presenteeism with HLPT (Pohling et al., 2016). We expect to find similar associations in our study (Fig. 1B). In sum, we propose the following four hypotheses:

- H1: (a) Mental job demands, physical job demands, responsibility, burdensome social environment and OID correlate positively and (b) job control, ease of replacement, and CSE correlate negatively with presenteeism (past 6 months).
- H2: (a) Mental job demands, physical job demands, and burdensome social environment correlate positively and (b) job control, ease of replacement, and CSE correlate negatively with HLPT.
- H3: (a) Mental job demands, physical job demands, and burdensome social environment correlate positively and (b) job control, responsibility, and CSE correlate negatively with absence duration (past 6 months).
- H4: (a) A burdensome social environment correlates positively and (b) job control, responsibility, and CSE correlate negatively with absence frequency (past 6 months).

### A Multilevel Perspective on Presenteeism

The above-mentioned associations are all located at the individual level of analysis though organizational identification already indicates the importance of higher order constructs in explaining individual behavior at work. Several studies adopting a multilevel perspective found that absence levels within work units influence individuals absence (Rentsch & Steel, 2003) and such relationships can be moderated by job-attitudes (Diestel et al., 2014). In this research, we acknowledge that the social *context* significantly affects organizational behavior (Johns, 2018). Context refers to situational and environmental stimuli that affect organizational behavior and its meaning by providing constraints and opportunities (Johns, 2006). Accordingly, contextual factors often need to be analyzed at a higher level of analysis than the actors they affect (Johns, 2018). Contextual effects often depend on individual characteristics and should therefore be analyzed in interaction with these.

Given these considerations, presenteeism can be expected to depend on the multi-level nature of organizations as well. Some authors have already noted the contextual factors’ importance for presenteeism. Johns (2010) discussed the relevance of presenteeism cultures and several theories on presenteeism, e.g., the social cognitive theory of presenteeism (Cooper & Lu, 2016) or the dialectical theory of presenteeism and absenteeism (Halbesleben et al., 2014), proposing that contextual variables affect psychological processes underlying attendance choices. In support, Dietz et al. (2020) found that leader presenteeism has a positive effect on employee presenteeism and sickness absence. Moreover, Luksyte et al. (2015) reported that coworker presenteeism can promote deviance and decrease engagement. In light of this increasing attention to contextual factors (Ruhle et al., 2020, see also Ferreira et al., 2019; Mach et al., 2018), a multi-level methodology is appropriate to advance our understanding of presenteeism. Therefore, we examine the unit-level context of attendance behavior (1) by considering preceding, objective unit-level absence as a predictor for presenteeism and absenteeism and (2) by conceiving presenteeism itself as a contextual factor. In the following, we outline the theoretical foundation of context and context strength that underlie our corresponding hypotheses.

### Context and Context Strength

We expect presenteeism and absenteeism context to affect individual attendance choices through implicit norms and behavioral convergence (Diestel et al., 2014). This assumption draws on social conformity theory (Asch, 1956), which acknowledges the effects of group norms and conformity pressures on individual behavior. Moreover, we expect that the *level* of contextual factors and their *consistency* affect individual behavior. The situational strength concept (Dalal & Meyer, 2012; Meyer et al., 2010; Mischel, 1977) suggests that a strong (highly consistent) context amplifies the relations of contextual factors and attenuates the relations of traits with individual behavior. Hence, situational strength can be defined as the presence of “implicit or explicit cues provided by external entities regarding the desirability of potential behaviors” (Meyer et al., 2010, p. 122). Similarly, the social information processing theory (Salancik & Pfeffer, 1978) emphasizes the role of perceived social context in reflecting socially acceptable attitudes and reasons for action as well as shaping expectations about individual behavior and its consequences. Especially research on organizational climate considered these ideas with “climate strength” as such a moderator (Schneider et al., 2013). In our study, we follow Chan’s (1998) additive and dispersion composition models for the statistical operationalization of context and context strength. Therefore, we use *means* of presenteeism and absenteeism within units to assess unit-level *context* (*additive* model) and their *standard deviations* to assess unit-level *context strength* (*dispersion* model) with lower standard deviations representing stronger contexts.

Following the situational strength concept, highly consistent absence contexts should be more likely to affect attendance behavior than weaker contexts. Accordingly (see Fig. 1A, B), we expect that individual absence duration and frequency are predicted by preceding unit-level absence duration and frequency and this relationship becomes stronger when context is strong (i.e., low unit-level absence dispersion).

In connection with presenteeism, two possible impacts of unit-level absence exist. First, the absence context and implied norms can create pressure to attend work despite sickness, when coworker absence is consistently low. Conversely, presenteeism might also be more prevalent in units with *high* absenteeism, since both types of attendance behavior are indicators of impaired health. To resolve these seemingly contradictory expectations, we examine preceding unit-level absence duration and frequency as different predictors for presenteeism. Since absence *duration* is usually understood as reflecting involuntary, health-related absence (Bakker et al., 2003), we propose that this variable is predicting presenteeism *positively*, as units with impaired health are characterized by both heightened absenteeism and presenteeism. In contrast, since absence *frequency* is usually viewed as a voluntary absence measure (Bakker et al., 2003), we propose that this variable is predicting presenteeism *negatively*, as units with less voluntary absence are likely to favor presenteeism through behavioral norms of low absence. Again, we expect that these relations are higher when absence context is stronger. Figure 1B summarizes our propositions. Of course, we acknowledge that interpreting absence duration/frequency as indicators for involuntary/voluntary absence is subject to controversy (Johns & Al Hajj, 2016). Nonetheless, we decided to employ this approach here, since the two absence indicators are not yet well studied in relation with presenteeism. Based on these considerations, we propose the following three hypotheses:

- H5: (a) Preceding absence duration (past 12–7 months) mean on the unit level positively predicts absence duration (past 6 months). Moreover, we predict (b) an interaction of preceding absence duration (past 12–7 months) mean and dispersion on the unit level in the sense that higher means of preceding absence duration relate more strongly to absence duration (individual level), when preceding absence duration dispersion is low (i.e., strong context).
- H6: (a) Preceding absence frequency (past 12–7 months) mean on the unit level positively predicts absence frequency (past 6 months). Moreover, we predict (b) an interaction of preceding absence frequency (past 12–7 months) mean and dispersion on the unit level in the sense that higher means of preceding absence frequency relate more strongly to absence frequency (individual level), when preceding absence frequency dispersion is low (i.e., strong context).
- H7: (a) Preceding absence duration (past 12–7 months) mean on the unit level is positively related and (b) preceding absence frequency (past 12–7 months) mean on the unit level is negatively related to self-reported presenteeism (past 6 months). Moreover, we predict two cross-level interactions: (c) preceding absence duration (past 12–7 months) mean and dispersion on the unit level interact in the sense that higher means of preceding absence duration relate more strongly to presenteeism when preceding absence duration dispersion is low (i.e., strong context), and (d) preceding absence frequency (past 12–7 months) mean and dispersion on the unit level interact in the sense that higher means of preceding absence frequency relate more strongly to presenteeism when preceding absence frequency dispersion is low (i.e., strong context).

Whereas these hypotheses examine context strength affecting context-behavior relationships, the following hypothesis examines a trait-behavior relationship as a context strength outcome. Therefore, we conceive unit-level presenteeism as a contextual factor affecting the relationship between trait and behavior at the individual level. More precisely, since strong contexts can attenuate dispositional effects on behavior, highly consistent presenteeism levels within units should decrease the relationship of CSE and presenteeism. In addition to the negative relationship between CSE and presenteeism (H1b), we propose a cross-level moderation of presenteeism context (Fig. 1A, B).

- H8: Increasing presenteeism context strength at the unit level (past 6 months) reduces the negative relationship of CSE and presenteeism (past 6 months) at the individual level.

### Attendance Behavior and Health

Absenteeism and presenteeism are health predictors with complementary roles (Caverley et al., 2007). The relationship of absenteeism with health impairment is well-examined. The meta-analysis of Darr and Johns (2008) reported small positive relations between sickness and absenteeism as well as between absence and subsequent sickness. Therefore, resource restoration by absenteeism appears limited since disrupted relationships or lower performance ratings might increase strain after return-to-work (Darr & Johns, 2008). In contrast, presenteeism is a stronger positive predictor of health impairment (Aronsson et al., 2011; Gerich, 2015). A proposed mechanism for this association is the lacking recuperation associated with continued attendance in times of sickness. In this sense, Demerouti and colleagues (2009) view presenteeism as an attempt to protect work resources when job demands are high. They propose that reduced recovery impairs health and makes it harder to deal with subsequent job demands, leading to a “loss spiral” since increased demands will in turn increase presenteeism even more.

While acknowledging these mutual relationships, our study sets a specific focus on viewing attendance behavior as a predictor for health (impairment). We hypothesize that the superiority of presenteeism vs. absenteeism in predicting health, which has been well understood on the individual level, holds true on the work unit level as well. Again, we conceive absence duration as a measure for involuntary, health-related absenteeism, and use it in contrast to presenteeism. Still, we also analyze absence frequency to justify this decision. As outcomes, we examine mental and physical health complaints since presenteeism relates to both (Gerich, 2015; Johns, 2010). Thus, we finally propose the following hypothesis (Fig. 1A, C):

- H9: Presenteeism (past 6 months) is a stronger positive predictor of physical and mental health complaints than absence duration (past 6 months). In addition, (a) individual presenteeism (past 6 months) is a stronger positive predictor of physical and mental health complaints than individual absence duration (past 6 months) and (b) the unit-level presenteeism mean (past 6 months) is a stronger positive predictor of physical and mental health complaints than the unit-level absence duration mean (past 6 months).

## Methods

### Participants and Procedure

We examined employees at a German DAX manufacturer’s plant and collected employee data in 11/2017 as part of a written survey. We ensured voluntary participation and the confidentiality of the responses. At this time, the workforce consisted of 3,004 employees, excluding interns, working students, and temporary workers. We assessed the membership of these employees to the 22 work units (*M*_*N*_ = 112, range: 17–422) with a multiple-choice survey item. 1,145 (38%) employees participated. After excluding *n* = 234 for statistical reasons (see “15”) the final sample size was 911 (*M*_Age_ = 41.06, *SD* = 11.33, 15% females, 47% blue-collar workers, 53% white-collar workers), working in 22 units (*M*_*N*_ = 41.41, range: 7–146). The absence management system in place required employees to inform their supervisor as soon as possible about a required sick leave. After three consecutive days of sick leave, employees had to provide a medical certificate of disability.

### Measures

The questionnaire assessed all variables with established instruments. Nearly all internal consistencies were acceptable (Table 1, α > 0.70, Nunnally & Bernstein, 1994, exception: job control). We averaged item scores to compute scale scores (exception: sum scores for the health instruments). In cases of single-item measures, we selected them from psychometrically tested scales in order to ensure reliability and validity (Fisher et al., 2016).

**Table 1:** Means, Standard Deviations, Internal Consistencies, and Pearson Correlations among Level-1 Variables

Variable	*M*	*SD*	α	1	2	3	4	5	6	7	8	9	10	11	12	13	14	15	16
1. Age	41.08	11.32		—															
2. Gender (0 = female; 1 = male)	0.85			.00	—														
3. Presenteeism (past 6 months)	2.72	1.10		–.10**	–.02	—													
4. Health-related lost productive time (HLPT)	2.16	4.18	.88^a^	.00	–.03	.27**	—												
5. Self-reported absence duration (past 6 months)	4.45	7.57		.02	–.06	.10*	.20**	—											
6. Self-reported absence frequency (past 6 months)	0.82	1.09		–.03	–.07*	.09*	.15**	.66**	—										
7. Mental job demands	2.81	0.54	.76	.06	.05	.17**	.10*	.04	.04	—									
8. Physical job demands	2.13	0.55	.83	–.11**	.16**	.23**	.16**	.15**	.07*	.18**	—								
9. Job control	3.27	0.80	.63	.11**	–.05	–.20**	–.17**	–.15**	–.06	–.16**	–.43**	—							
10. Ease of replacement	3.17	1.12		.01	–.02	–.11**	–.11**	.05	–.04	–.16**	.01	.08*	—						
11. Responsibility	3.89	0.95		.13**	.12**	–.03	–.08	–.05	–.02	.10**	–.07*	.26**	–.07*	—					
12. Burdensome social environment	2.61	0.79	.71	–.07*	.11**	.23**	.16**	.09**	.03	.23**	.28**	–.26**	–.16**	–.01	—				
13. Core self-evaluations	3.72	0.53	.81	–.05	–.01	–.23**	–.29**	–.14**	–.10**	–.30**	–.23**	.28**	.09**	.21**	–.25**	—			
14. Organizational identification	3.18	1.03	.82	.11**	.02	–.03	–.10**	–.11**	–.07	–.02	–.16**	.18**	.01	.25**	–.10**	.30**	—		
15. Physical health complaints	18.62	14.06	.93	.05	–.03	.35**	.42**	.20**	.18**	.28**	.39**	–.26**	–.10**	–.06	.32**	–.48**	–.16**	—	
16. Mental health complaints	12.88	9.92	.93	.02	.00	.30**	.37**	.15**	.13**	.30**	.26**	–.23**	–.14**	–.03	.33*	–.61**	–.15**	.75**	—

*N* = 911 employees nested within 22 work units. The above statistics for absence variables and HLPT were computed before square root transformation

^a^ Internal consistency for the HLPT instrument’s performance limitation subscale

^*^ *p* < .05. ** *p* < .01

#### Work-related and Personal Factors

*Mental job demands* were assessed using the six-item scale “excessive demands” (e.g., “There are things to do, for which one is neither sufficiently educated nor prepared.”) of the German Salutogenetic Subjective Work Analysis (SALSA; Rimann & Udris, 1997). All items of this questionnaire are rated on a 5-point Likert scale from 1 (“strongly disagree”) to 5 (“strongly agree”).

*Physical job demands* were measured using the scale “physical demands” of the German Questionnaire for Subjective Assessment of Stress Factors at the Workplace (FEBA; Slesina, 2009). It lists 14 factors like “adverse body posture” or “carrying heavy loads”, the frequency of which is rated on a 4-point scale ranging from 1 (“never”) to 4 (“often”).

*Job control* was assessed using the respective scale in the SALSA questionnaire (see above). It comprises three items (e.g., “This work allows making a many autonomous decisions.”).

*Ease of replacement* was assessed using the item “When I am absent from work for up to a week, someone else can fill in for me easily.” (Johns, 2011) with a 5-point Likert scale from 1 (“strongly disagree”) to 5 (“strongly agree”).

We assessed the perceived job *responsibility* with the item “My work requires great responsibility.” (SALSA questionnaire, scale “qualification requirements and responsibility”).

*Burdensome social environment* was assessed using the equally named scale of the SALSA questionnaire, which includes three items on coworker-related stress factors (e.g., “Often there are tensions at the workplace.”).

*Core self-evaluations* were assessed using the German version (Weiherl, 2007) of the Core Self-Evaluations Scale (CSES; Judge et al., 2003). The scale’s 12 items (e.g., “I am confident I get the success I deserve in life.”) are rated on a 5-point Likert scale from 1 (“strongly disagree”) to 5 (“strongly agree”).

*Organizational identification (OID)* was assessed using three items (e.g., “I am very interested in what others think about [name of company].”) from Mael and Ashforth’s (1992) with responses ranging from 1 (“strongly disagree”) to 5 (“strongly agree”).

##### Attendance Behavior and HLPT

*Presenteeism (past 6 months)* was assessed using the item “How often have you attended work in the last six months despite feeling sick?” (response format: 1 = ‘never’ to 5 = ‘almost always’). This phrasing and single-item approach is a common but response formats vary (Ruhle et al., 2020). Here we used a subjective approach to ensure demarcation from the HLPT measure (cf. below), which already requires indicating days of presence and sickness in the last 14 days. The recall period of the past six months was chosen to equal the one used in the self-reported absenteeism measures, thus, improving comparability. To analyze presenteeism at the unit level, we computed means and standard deviations for each work unit (see procedure with the absence data below).

*Health-related lost productive time (HLPT)* due to health complaints was assessed using the German HLPT questionnaire (Weiherl, 2007; similar to Koopman et al., 2002). It assesses four components that are integrated into a single value (HLPT). First, participants indicate the number of days they spent at work in the last two weeks. Second, they specify the number of days they spent at work while suffering from health complaints. Participants continue with the questions if they reported at least one day. For the third component, respondents indicate the typical duration of their health complaints (11-point scale from 0 = ‘not at all’ to 10 = ‘all day’). The fourth component assesses eight performance limitation (e.g., work quality, concentration) caused by health impairments with 11-point Likert scales (0 = ‘strongly disagree’ to 10 = ‘strongly agree’), which are transformed to percentages. The four components are aggregated by multiplication of (1) the proportion of attendance days with health impairments in relation to attendance days, (2) the typical duration of health impairments, (3) the extent of performance limitation, and (4) a reference value specifying the weekly working time (here: 35 h).

*Self-reported absence duration and frequency (past 6 months)* were measured using two customized items: “How many work days have you been absent due to sickness in the last six months?” (*absence duration*) and “In how many absence periods did these days fall? The number of days is irrelevant here; one absence period can last for three days, two weeks or even six weeks.” (*absence frequency*). As recommended (Johns, 1994), we used introductory phrases that weaken the connotation of absenteeism as deviant behavior and presented a free response format (number field) with regard to the six months before participation. In order to reduce the known tendency of underreporting absence, we explicitly probed for *sickness* absenteeism (Johns & Miraglia, 2015).

Archival data on *objective absence duration and frequency* were provided by the company for two timeframes. The first timeframe was 11/2016–04/2017 (“past 12–7 months”) covering a six-months-span before the self-reported absence measure’s six-month recall period, the second was 05/2017–10/2017 (“past 6 months”) and corresponded to the self-reported absence measure’s recall period. The datasets contained 4,397 (past 12–7 months) and 3,163 (past 6 months) absence events. The absence data only included periods of leave that were due an employee’s own sickness as indicated by either the person herself (if absence duration < 3 days) or attested by a physician (if absence duration ≥ 3 days). We aligned the assignment of work units in the absence dataset with the units addressed in the questionnaire. We aggregated absence data from smaller subunits to the corresponding higher-level units and excluded units not mentioned in the survey.^1^ Next, we aggregated absence events (i.e., sum of absence days and periods) within persons resulting in 1,801 (past 12–7 months) and 1,476 (past 6 months) cases. We supplemented unrecorded cases of employees without absence resulting in final data of 2,407 (past 12–7 months) and 2,466 (past 6 months) cases. The mean and standard deviation of absence duration and absence frequency was calculated for each work unit (Chan, 1998). Analyses revealed less absenteeism (duration and frequency) in the second than in the first timeframe, which might reflect seasonal absence variations (Léonard et al., 1990). In line with Johns (1994), objective and self-reported absence correlated positively at the unit level (past 6 months: *r*_Duration_ = 0.58, *r*_Frequency_ = 0.43).

##### Physical and Mental Health

*Physical health complaints* were assessed with the Giessen Subjective Complaints List (GBB-24; Brähler & Scheer, 1995). Employees rate the intensity of 24 complaints during past two weeks (0 = ‘no complaints’ to 4 = ‘strong complaints’) relating of four subscales (exhaustion, gastrointestinal, cardiovascular, and musculoskeletal complaints).

*Mental health complaints* were assessed using the shortened German Depression Anxiety Stress Scales (Emmermacher, 2009; Lovibond & Lovibond, 1995). It comprises three subscales (depression: “I felt that life was meaningless”, anxiety: “I felt scared without any good reason”, and stress: “I found it difficult to relax”) with seven items each, asking for the frequency of complaints during the past two weeks (0 = ‘never’ to 3 = ‘very often’).

##### Control Variables

Age and gender correlate with attendance behavior (Miraglia & Johns, 2016; Ng & Feldman, 2008; Scott & McClellan, 1990). Therefore, we included both as level-1 control variables. We assessed work unit size as control variable at level 2, since it relates to health (Zwingmann et al., 2014).

#### Data Processing and Analysis

We used R 3.4.4 (processing objective absence data), HLM 6.06 (multilevel analyses), and SPSS 25.0 (remaining analyses) for statistical computing. For the self-reported absence variables, we trimmed 26 implausible scores. Following Field (2009), we trimmed scores with more extreme z-values than ± 3.29 by setting them to these respective threshold values.

##### Missing Data

About 3% of all values were missing, 616 cases had at least one missing value. Missing values were not completely at random (Little, 1988; MCAR; χ^2^ = 58,752.70, *df* = 53,484, *p* < 0.01). To achieve appropriate means and standard errors, we applied multiple imputation at the item-level (Eekhout et al., 2014) with five imputed datasets (Schafer & Graham, 2002) using fully conditional specification. Constraints were set to prevent imputation outside of the bounds of response scales (e.g., an imputed value for a variable with a 5-point scale couldn’t be lower than 1 or higher than 5). As imputation of categorical variables is not supported in SPSS 25, we followed Allison (2005) and removed 113 cases. We pooled results among datasets using the program packages or with a manual approach (Rubin, 2004).

##### Normal Distribution of the Variables

Considering our large sample, we used the criteria of skewness < 2 and kurtosis < 5 to test normality (Hammer & Landau, 1981). We found violations for all self-reported and objective absence variables and HLPT, as can be expected, since these variables represent count data. We subjected these variables to square root transformation (Clegg, 1983), after which they fulfilled the above criteria for normality.

##### Statistical Analyses and Final Sample Size

We tested hypotheses with hierarchical linear modeling (HLM; Bryk & Raudenbush, 1992). Even though our hypotheses included bivariate relations, we decided to follow a regression approach in order to control for potential confounding variables (O'Neill et al. 2014). In total, 911 cases remained for analysis (113 cases were removed because an imputation of categorical variables was not supported in SPSS 25; see Allison, 2005; 121 cases indicated a residual work unit “other” so that the work unit was unclear). We z-standardized variables as grand mean centering reduces multicollinearity problems, controls for level-1 variables when examining level-2 effects (Hofmann & Gavin, 1998), and to achieve comparable parameter estimates. Level-2 interaction terms were standardized before multiplication. To obtain unbiased estimates regarding the proposed cross-level interaction, the level-1 variable CSE was standardized within groups (Hofmann & Gavin, 1998). For testing H9, level-1 presenteeism was standardized within groups to isolate its within-group variation, whereas between-group variation was already accounted for with the level-2 predictor (Hofmann & Gavin, 1998).

## Results

Table 1 and 2 show descriptive statistics and correlations, Table 2 additionally reports intra-class correlations (ICC, Bliese, 2000). ICC(1) estimates the variance in individual scores explained by group membership. Median values are about 0.12 in organizational research (James, 1982). The ICC(2) estimates the group means reliability (Bliese, 2000). Glick (1985) suggested 0.60 as minimum value. Based on these values, the intra-class correlations here appeared high enough to justify unit-level aggregation and further analyses.

**Table 2:** Means, Standard Deviations, Intra-Class Correlations, and Pearson Correlations among Level-2 Variables

Variable	*M*	*SD*	ICC(1)	ICC(2)	1	2	3	4	5	6	7	8	9	10	11	12
1. Work unit size (12 months prior to survey)	107.55	107.09			—											
2. Work unit size (at time of survey)	112.09	90.46			.92**	—										
3. Presenteeism (past 6 months): mean	2.68	0.34	0.05	0.66	.14	.13	—									
4. Presenteeism (past 6 months): dispersion	1.07	0.17			.00	.02	.54*	—								
5. Objective absence duration (past 12–7 months): mean	9.68	5.15	0.10	0.82	.02	.33	.18	.02	—							
6. Objective absence duration (past 12–7 months): dispersion	15.81	9.01			.14	.42*	.25	–.08	.91**	—						
7. Objective absence frequency (past 12–7 months): mean	1.49	0.32	0.11	0.83	.05	.14	–.34	–.14	.54**	.29	—					
8. Objective absence frequency (past 12–7 months): dispersion	1.39	0.28			.23	.12	–.22	–.28	–.10	.12	.01	—				
9. Objective absence duration (past 6 months): mean	5.94	2.43	0.07	0.76	.33	.37	.05	–.08	.50*	.41	.46*	.03	—			
10. Objective absence duration (past 6 months): dispersion	10.84	4.72			.23	.35	–.13	–.32	.52*	.54*	.39	.16	.82**	—		
11. Objective absence frequency (past 6 months): mean	1.11	0.24	0.04	0.62	.24	.15	–.35	–.15	.17	.04	.56**	.07	.69**	.38	—	
12. Objective absence frequency (past 6 months): dispersion	1.30	0.24			.21	.22	–.32	–.08	.17	.08	.33	–.14	.49*	.28	.79**	—

*N* = 22. The above statistics for absence variables were computed before square root transformation

^*^ *p* < .05. ** *p* < .01

Moreover, we run a confirmatory factor analysis (MLR estimation) in R to examine if all work-, person-, and health-related variables that we assessed with multi-item measures (i.e., mental and physical demands, job control, organizational identification, burdensome social environment, core-self evaluations, physical and mental health complaints) represent distinct constructs. This assumption was confirmed since the eight-factor model-fit was acceptable (RMSEA = 0.059, 90%CI [0.058, 0.060]; SRMR = 0.066; Schermelleh-Engel et al., 2003). Model chi-square was 14,759.019 (*df* = 3,541, *p* < 0.01), CFI was 0.728, and TLI was 0.719. Moreover, results of the correlation analyses (Table 1) further supported construct distinctiveness. Out of these many interrelationships, only three were higher than *r* > 0.50 (two based on conceptual relatedness; absence frequency-absence duration with *r* = 0.66, physical and mental health complaints with *r* = 0.75, and core-self evaluations-mental health complaints with *r* = –0.61).

### Individual Level Predictors of Attendance and Absence

Results in Table 3 (upper part) reveal diverging relationships between the predictors and presenteeism, HLPT, and absence duration and frequency. Considering the *control variables*, men reported smaller HLPTs, absence durations, and absence frequencies, while presenteeism was not associated with gender. Age related negatively to presenteeism.

**Table 3:** Results of Hierarchical Linear Modeling Analyses for Work-related Factors, Individual Factors and Unit-Level Attendance Behavior as Predictors of Attendance Behavior and HLPT (Hypotheses 1 to 7)

	Presenteeism (past 6 months)		HLPT		Self-reported absence duration (past 6 months)		Self-reported absence frequency (past 6 months)	
Model component	Coefficient	*SE*	Coefficient	*SE*	Coefficient	*SE*	Coefficient	*SE*
Intercept	0.04	0.07	–0.01	0.04	0.03	0.04	0.10	0.06
Level 1 variables								
Age	–0.08*	0.04	0.02	0.03	0.01	0.04	–0.03	0.03
Gender (0 = female, 1 = male)	–0.07	0.04	–0.10**	0.03	–0.10**	0.03	–0.11**	0.03
Mental job demands	0.06	0.04	0.00	0.04	–0.01	0.04	0.01	0.04
Physical job demands	0.11*	0.04	0.10*	0.04	0.08*	0.04	0.05	0.04
Job control	–0.09	0.04	–0.09*	0.04	–0.07	0.04	–0.02	0.04
Ease of replacement	–0.07*	0.04	–0.09*	0.04	0.02	0.03	–0.04	0.03
Responsibility	0.03	0.04	0.04	0.04	0.02	0.04	0.01	0.04
Burdensome social environment	0.11**	0.04	0.09*	0.04	0.02	0.04	–0.01	0.04
Core self-evaluations	–0.16**	0.04	–0.24**	0.04	–0.06	0.04	–0.05	0.04
Organizational identification	0.07*	0.03	–0.03	0.03	–0.06	0.04	–0.04	0.04
Level 2 variables								
Work unit size	–0.02	0.04	0.01	0.03	0.00	0.03	–0.02	0.03
Objective absence duration mean (past 12–7 months)	0.56	0.29			0.16*	0.06		
Objective absence duration dispersion (past 12–7 months)	–0.22	0.14			–0.11	0.05		
Objective absence duration mean × dispersion (past 12–7 months)	–0.01	0.03			–0.03	0.03		
Objective absence frequency mean (past 12–7 months)	–0.54*	0.19					0.00	0.08
Objective absence frequency dispersion (past 12–7 months)	0.04	0.13					–0.12	0.10
Objective absence frequency mean × dispersion (past 12–7 months)	0.04	0.07					0.09	0.07
Model properties								
ICC(1) of outcome	0.04		0.02		0.01		0.01	
R^2^_Level 1_	11.51%		13.64%		4.37%		2.76%	
R^2^_Level 2_	99.92%		94.38%		98.35%		0.00% ^1^	

*N* = 911 employees nested within 22 work units. Standardized coefficients are reported

^1^ The residual variance component at level 2 was estimated higher in the fit model than in the random-intercept-only model, which indicates that the latter, simpler model fits

the data better than the former does. Since this pattern would lead to a negative R^2^_Level 2_, the coefficient was set equal to zero instead

^*^p < .05. **p < .01

Significant *work-related* and *individual variables* associated with higher *presenteeism* were higher physical job demands, lower ease of replacement, higher burdensome social environment, lower CSE, and higher OID, supporting H1. Mental job demands, responsibility, and job control (γ = –0.09, *p* = 0.053) failed to reach significance. In sum, H1 has to be rejected. However, many of its components are supported by our analyses.

Regarding *HLPT*, we found significant effects for higher physical job demands, lower job control, lower ease of replacement, higher burdensome social environment, and lower CSE, which support H2. However, responsibility, mental job demands, and OID were no significant predictors. Again, H2 as a whole has to be rejected whereas some of its components are supported by our analyses.

Regarding *self-reported absence duration* and *frequency* almost no predictors reached significance, an exception being higher physical job demands which related to higher absence duration (in line with H3), but not frequency. Still, both H3 and H4 have to be rejected.

### Unit-Level Predictors of Attendance and Absence

Table 3 (lower part) shows results concerning relations of unit-level predictors with employees’ attendance and absence behavior. At the unit level, preceding *absence duration* was a significant positive predictor for self-reported absence duration, supporting H5a. In contrast, no relationship emerged between preceding unit-level *absence frequency* and self-reported absence frequency, therefore rejecting H6a. Furthermore, we found no interaction effects of mean and dispersion with regard to either of the two absence variables. Accordingly, H5b and H6b are not supported by the data.

Considering individual *presenteeism* predictors at the unit-level, objective absence duration mean (past 12–7 months) failed to reach significance (γ = 0.56, *p* = 0.08), contrasting with H7a. However, objective absence frequency mean (past 12–7 months) was significantly negatively related, supporting H7b. The interactions of unit-level absence mean and dispersion in context with presenteeism were non-significant regarding absence duration and frequency. Thus, H7c and H7d are not supported by the data.

### Context Strength as Moderator for Relationships between CSE and Presenteeism

**Fig. 2:**
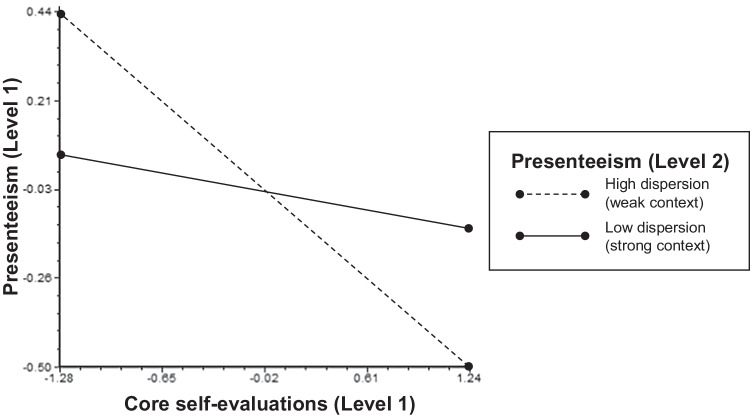
Cross-level Interaction of Unit-Level Presenteeism Dispersion and Individual Core Self-Evaluations with the Outcome of Individual Presenteeism. *Note*. “High” and “Low” values for unit-level presenteeism dispersion reflect the average of the upper and lower quartiles

We proposed that presenteeism context strength at the unit level attenuates the negative relationship of CSE and presenteeism at the individual level. Multilevel analysis results for presenteeism at level-1 (ICC_1_ = 0.04, *R*^2^_Level 1_ = 6.06%, *R*^2^_Level 2_ = 24.15%) revealed the expected cross-level interaction of CSE and the dispersion of unit-level presenteeism (γ = –0.15, *p* < 0.01) after adjusting for age (γ = –0.09, *p* < 0.05), gender (γ = –0.01, *p* > 0.05), CSE (γ = –0.22, *p* < 0.01), and work unit size (γ = 0.05, *p* > 0.05). As depicted in Fig. 2, the negative, individual-level relationship of CSE and presenteeism was lower when unit-level presenteeism dispersion was low (strong context) than when it was high (weak context). This supports H8.

### Attendance and Absence Behavior in Relation to Health

Results in Table 4 address H9. We calculated two models for both (physical and mental) health complaints. The first included control variables and absenteeism as predictors while the second added individual and unit-level presenteeism. Regarding the control variables, only age related positively to health complaints.

**Table 4:** Results of Hierarchical Linear Modeling Analyses for Presenteeism and Absenteeism as Predictors of Health Complaints (Hypothesis 9)

	Physical health complaints				Mental health complaints			
	Model 1.1 Absenteeism only		Model 1.2 Absenteeism and presenteeism		Model 2.1 Absenteeism only		Model 2.2 Absenteeism and presenteeism	
Model components	Coefficient	*SE*	Coefficient	*SE*	Coefficient	*SE*	Coefficient	*SE*
Intercept	–0.07	0.05	–0.06	0.04	–0.04	0.05	–0.03	0.04
Level 1 variables								
Age	0.07*	0.03	0.11**	0.03	0.03	0.03	0.06*	0.03
Gender (0 = female, 1 = male)	–0.03	0.03	–0.02	0.03	0.01	0.03	0.02	0.03
Self-reported absence duration	0.16*	0.06	0.14*	0.06	0.14*	0.06	0.11	0.06
Self-reported absence frequency	0.03	0.06	0.02	0.06	0.00	0.06	0.00	0.06
Presenteeism			0.30**	0.03			0.25**	0.03
Level 2 variables								
Work unit size	0.01	0.05	0.01	0.03	–0.01	0.05	–0.01	0.03
Objective absence duration mean (past 6 months)	0.28*	0.11	0.13	0.08	0.19	0.10	0.07	0.08
Objective absence frequency mean (past 6 months)	–0.21	0.11	–0.05	0.07	–0.14	0.10	–0.03	0.07
Presenteeism mean (past 6 months)			0.23**	0.04			0.22**	0.04
Model properties								
ICC(1) of outcome	0.05		0.05		0.03		0.03	
R^2^_Level 1_	4.59%		14.07%		2.03%		9.45%	
R^2^_Level 2_	47.44%		99.84%		38.28%		99.86%	

*N* = 911 employees nested within 22 work units. Standardized coefficients are reported. *p < .05. **p < .01

Regarding *physical health complaints*, absence duration had a significant positive coefficient as a self-reported, level-1 predictor and as an objective, unit-level predictor. In contrast, absence frequency did not predict physical complaints at either level of analysis. When adding presenteeism to this model, it served as a significant predictor as a level-1 variable and as a unit-level variable. Moreover, unit-level absence duration was no longer significant while level-1 absence duration retained its significance.

We found a similar pattern in models predicting *mental health complaints*. However, the only absence-related predictor was self-reported absence duration in the model without presenteeism (Model 2.1) while unit-level absence failed to predict mental health complaints. When adding presenteeism to the model, none of the absence variables at either level of analysis were significant. However, individual and unit-level presenteeism were significant positive predictors. In sum, we found support for H9a and H9b.

## Discussion

In our multilevel study, we examined individual and unit-level factors affecting presenteeism, absenteeism, and health-related productivity losses (HLPT). We considered “presenteeism context strength” as a unit-level predictor and examined its influence on the individual-level relationship between CSE and presenteeism. Moreover, we assessed the diverging relations of absenteeism and presenteeism with health at the individual and work unit level.

### Theoretical Implications

We found that multiple work-related and individual factors were associated with presenteeism and HLPT in accordance with our hypotheses, while surprisingly few related to absence duration or frequency. For presenteeism and HLPT, level-1 predictors were mostly similar. Physical demands and ease of replacement related to both variables, which is in line with other studies (Johns, 2011; Miraglia & Johns, 2016). We found similar results for job control, which were, however, only significant for HLPT, and burdensome social environment. The latter effect can be expected given the negative relation of collegial support and presenteeism (Miraglia & Johns, 2016). A burdensome social environment did not seem to relate to absence in the context of our model. We assume that conflictual relationships with colleagues do not promote withdrawal from work, but increase effort investment by means of presenteeism, which comes at the cost of decreased productivity due to health impairments. CSE related negatively to presenteeism and HLPT, supporting its importance here (Johns, 2010, 2011). Employees with high CSE may cope well with high job demands and delayed work after sick leave due to their internal locus of control and higher self-efficacy, also decreasing the perceived pressure to attend work despite sickness. However, CSE did not relate to absenteeism. Likely this is because CSE is positively associated with health (Tsaousis et al., 2007), thus, reducing the confrontation with the choice whether to work when sick. As expected, we found that organizational identification increased presenteeism, but did not affect HLPT. This pattern supports the idea that certain types of presenteeism are a positive behavior under specific conditions, e.g., in terms of organizational citizenship behavior (Ruhle et al., 2020).

Neither responsibility nor mental job demands related to presenteeism and HLPT in our data. Our broad measure of perceived job responsibility might be a limitation here. Future research could employ other measures related to the hierarchical level (Djurdjevic et al., 2017). The missing relationships with mental job demands contradict meta-analytic results (Miraglia & Johns, 2016) but might be caused by considering more specific and correlated variables (e.g., replaceability) reducing incremental variance for mental job demands.

We identified few significant predictors affecting absence duration and frequency. This is common (Caverley et al., 2007) because self-reported work and personal characteristics relate more strongly to presenteeism (Miraglia & Johns, 2016). In this study, gender and physical job demands were the only level-1 variables relating to self-reported absence; the latter predictor was only associated with absence duration and not frequency. These findings reveal that presenteeism serves as a more effective target for health promotion programs than absenteeism (Miraglia & Johns, 2016), since it is more strongly affected by work-related factors. At the unit level, as expected, objective absence indicators (past 12–6 months) predicted presenteeism (past 6 months) in a diverging pattern. Higher absence frequencies preceded less presenteeism whereas the expected positive relation between absence duration and presenteeism failed to reach statistical significance. Context strength did not moderate these relations as hypothesized, however, this unexpected result can be challenged in future research by using other methodological approaches as proposed below. Nonetheless, our findings provide insight into the temporal association between the absence context and individual presenteeism by linking objective absence and presenteeism in a longitudinal way. On the other hand, the unit-level predictors for individual absence were rather weak, with only preceding objective absence duration predicting self-reported absence duration (we found no such relation for absence frequency). This means that the unit-level context more strongly affects absence duration than absence frequency. As we found no moderating effect of context strength regarding the two absence variables, this could indicate that the situational strength concept in terms of absence cultures was not relevant in our sample.

In contrast, presenteeism context strength moderated the relationship between CSE and presenteeism. In line with the situational strength concept (Mischel, 1977), low dispersion levels of presenteeism within units (strong contexts) attenuated the negative relationship of CSE and presenteeism at the individual level. Thus, our results reveal that presenteeism context strength has the potential to shape such a trait-presenteeism relationship and support the need to consider organizational contexts and behaviors in conjunction (Johns, 2018).

We further found that presenteeism serves as a better predictor for physical and mental health than absenteeism at the individual and the unit level, because unit-level absenteeism is no longer a significant predictor once unit-level presenteeism is included in the model. This supports prior results (Aronsson et al., 2011) and extends them by using a multilevel approach. Unit-level presenteeism likely exerts its influence on health through other pathways than individual presenteeism, for instance, through behavioral convergence with the social context (Salancik & Pfeffer, 1978) that favor high presenteeism, or through contagion in units with many sick employees present at work (Lovell, 2004). Moreover, absence duration but not absence frequency predicted health. This fits to the understanding of absence duration as an indicator for involuntary (i.e., health-related) absenteeism and absence frequency as reflecting rather voluntary (i.e., motivation-related) absenteeism (Bakker et al., 2003). Our results further support the notion that presenteeism and absenteeism have *complementary* roles in predicting health (Gerich, 2015).

### Limitations

Our results are not without limitations. First, the number of groups was limited which reduces statistical power (Scherbaum & Ferreter, 2009). However, average group size was comparably high, which is more important when examining cross-level interactions (Mathieu et al., 2012). Second, the ICC(1)-values were low for some variables limiting variance explained by unit membership. A cause might be the strong similarity of the groups that were situated at the same location in one company or data aggregation at a level to high, masking differences among smaller subunits. Third, the largely cross-sectional design limits the interpretability of temporal relevance and causal direction. However, we used preceding objective unit-level absence, thus, adding a longitudinal component, and consecutive reference timeframes in the items, which built a conceptual temporal order among variables.

Fourth, the results on absenteeism marked by few significant predictors need to be put into context. We note that zero-order correlations of some variables with absence duration and frequency are in fact significant but lose relevance in the multilevel analyses. This could be, of course, a consequence of the additional variables that are controlled for in this type of analysis. Moreover, we have to consider that we conducted a nonlinear transformation of the absence data in order to fulfill the requirement for using hierarchical linear modeling. We are aware that some authors use more advanced regression models to deal with skewed absence data, such as negative binomial regression (for an overview, see Becker et al., 2019). However, such approaches are not yet available for multi-level modeling. Thus, a square root transformation seemed both more feasible and appropriate in the context of our study, also because our initial analyses without square root transformation did not show vastly differing results. The non-significant level-2 predictors can be explained by the small ICCs of the self-reported absence variables, limiting the variance to be explained.

Finally, our presenteeism measure is highly subjective. Showing presenteeism “frequently” might mean something different for different employees. However, using more clearly defined frequency anchors is also problematic (Schwarz et al., 1985). Alternatively, researchers might ask respondents about the amount of days they spent at work while sick in a free response format (number field), which could improve the comparability with other absence measures. However, presenteeism days may not be as clearly countable or available in memory like absence days and even relate to diverging predictors (Johns, 2011).

### Future Research Directions

Future studies on attendance behavior should continue to consider presenteeism and absenteeism in conjunction. A stronger focus on contextual factors can prove fruitful since the work-related and personal factors that shape attendance behavior likely vary across different contexts. For instance, job insecurity might relate stronger to presenteeism when unemployment rates are high (Hansen & Andersen, 2008). Moreover, adequate internal (i.e., CSE) and external (i.e., job control) resources might help maintaining performance when health is impaired (Karanika-Murray & Biron, 2020). Currently, the influences of the COVID-19 pandemic on (virtual) sickness presenteeism while working from home are also being investigated (Ruhle & Schmoll, 2021). This needs further exploration.

Additionally, consequences of the organization’s economic situation such as downsizing (Caverley et al., 2007) might affect the relevance of certain factors promoting presenteeism. Future studies should identify relevant contextual variables (e.g., presenteeism culture and absence context; Dew et al., 2005; Rentsch & Steel, 2003) and individual differences (e.g., guilt proneness; Schaumberg & Flynn, 2017) and explore situational strength effects and moderating factors (Johns, 2018).

Alternative methods for assessing the unit-level presenteeism context may be evaluated in future research. A promising approach is the referent-shift consensus (Chan, 1998), which uses questionnaire items that refer to the organizational units to which the responses are aggregated. It will be interesting to see if higher agreement levels will be obtained with this method since presenteeism is likely a less overt and discernible behavior than absenteeism, making it harder for individuals to assess its prevalence within units.

Future studies could further examine how self-reports of presenteeism compare to perceived presenteeism norms within teams or units. Corresponding absenteeism research has revealed that 85% to 95% of employees indicate being less absent than their colleagues (“Lake Wobegon effect”; Harrison & Shaffer, 1994). When examining such effects with regard to presenteeism, the results may not be as straightforward since the social desirability of this behavior might be different across teams or work units. While elevated absence is considered a deviant behavior (Johns, 1994), presenteeism can be viewed differently by employees and supervisors, either as a risk factor for health and performance or as a sign of organizational commitment. Consequently, diverging patterns of over- and underreporting could arise in different groups because of different norms regarding presenteeism.

Another interesting idea for future research is to explore through which pathways unit-level absenteeism vs. unit-level presenteeism affects individual behavior because presenteeism is not always visible, as opposed to absenteeism. We know of no reliable data or theory on this question and believe that the (perceived) visibility of presenteeism and absenteeism could be an important moderator variable influencing both the phenomenon of unit level presenteeism and absenteeism as such and effects on individual behavior grounded in unit-level absenteeism or presenteeism. Finally, it also has to be mentioned that other research on presenteeism is focusing on potential *positive* effects of this behavior for the “presentee” (Dew et al., 2005; Karanika-Murray & Biron 2020; Ruhle & Süß, 2020). For example, it can be argued that this behavior balances health demands and performance demands and that not only dysfunctional but also functional forms of presenteeism exist. Our findings show that such differentiations are warranted. In our study, we have found that a strong identification with the organization is positively associated with presenteeism. Future studies should seek to discover potential positive antecedents and outcomes of presenteeism in order to inform both the theoretical understanding and practical interventions targeted at reducing dysfunctional forms of presenteeism.

### Practical Implications

Our study results have important implications for human resource and occupational health management. In contrast to direct assessments, data on absenteeism might be an inexpensive alternative identifying work units with risks of developing high presenteeism, especially those with *low absence frequency*. Such units could then be targeted with health promotion programs which affect presenteeism much stronger than absenteeism (Miraglia & Johns, 2016). Moreover, human resource managers should consider contextual factors when aiming to reduce presenteeism since we found unit-level presenteeism context strength affecting the relationship between CSE as health predictor and presenteeism. Therefore, person-centered approaches for improving employees’ health will have a limited effect in reducing presenteeism under situations of high contextual consistency and strong behavioral norms. Finally, the superiority of presenteeism as opposed to absenteeism in predicting employees’ health underlines its crucial role for occupational health management. Consequently, the narrow focus on absenteeism in conventional approaches of occupational health is once more called into question.

## Conclusions

In conclusion, our results highlight the individual and work-unit variables associated with attendance behavior and reveal that presenteeism context strength has the potential to shape trait-presenteeism relationships. Moreover, unit-level presenteeism appears to be a better predictor for individual health than unit-level absenteeism. Overall, this study demonstrates the value of a contextual, multilevel approach in the pursuit to understand presenteeism and absenteeism at work.

## Data Availability

The data that support the findings of this study are available from Siemens Energy Global GmbH & Co KG but restrictions apply to the availability of these data, which were used under licence for the current study, and so are not publicly available. Data are however available from the authors upon reasonable request and with permission of Siemens Energy Global GmbH & Co KG.

## Footnotes

1 Of course, single employees may have changed work units over time. Unfortunately, this was not documented so that we could not analyze potential changes. However, the work units were not restructured and the organization reported that such changes were very rarely because units worked continuously and stable during this time.
